# Bathymetric evolution of black corals through deep time

**DOI:** 10.1098/rspb.2023.1107

**Published:** 2023-10-04

**Authors:** Jeremy Horowitz, Andrea M. Quattrini, Mercer R. Brugler, David J. Miller, Kristina Pahang, Tom C. L. Bridge, Peter F. Cowman

**Affiliations:** ^1^ Australian Research Council Centre of Excellence for Coral Reef Studies, James Cook University, 101 Angus Smith Drive, Townsville, Queensland 4811, Australia; ^2^ Biodiversity and Geosciences Program, Museum of Tropical Queensland, Queensland Museum, 70-102 Flinders street, Townsville, Queensland 4810, Australia; ^3^ Department of Invertebrate Zoology, National Museum of Natural History, Smithsonian Institution, 10th street and Constitution avenue North West, Washington, DC 20560, USA; ^4^ Department of Natural Sciences, University of South Carolina Beaufort, 1100 Boundary Street, Beaufort, SC 29902, USA; ^5^ Division of Invertebrate Zoology, American Museum of Natural History, Central Park West at 79th Street, New York, NY 10024, USA; ^6^ Centre for Tropical Bioinformatics and Molecular Biology, Molecular and Cell Biology, James Cook University, 101 Angus Smith Drive, Townsville, Queensland 4811, Australia; ^7^ College of Science and Engineering, James Cook University, 101 Angus Smith Drive, Townsville, Queensland 4811, Australia

**Keywords:** ultraconserved elements, antipatharia, shelf, slope, abyss, adaptations

## Abstract

Deep-sea lineages are generally thought to arise from shallow-water ancestors, but this hypothesis is based on a relatively small number of taxonomic groups. Anthozoans, which include corals and sea anemones, are significant contributors to the faunal diversity of the deep sea, but the timing and mechanisms of their invasion into this biome remain elusive. Here, we reconstruct a fully resolved, time-calibrated phylogeny of 83 species in the order Antipatharia (black coral) to investigate their bathymetric evolutionary history. Our reconstruction indicates that extant black coral lineages first diversified in continental slope depths (∼250–3000 m) during the early Silurian (∼437 millions of years ago (Ma)) and subsequently radiated into, and diversified within, both continental shelf (less than 250 m) and abyssal (greater than 3000 m) habitats. Ancestral state reconstruction analysis suggests that the appearance of morphological features that enhanced the ability of black corals to acquire nutrients coincided with their invasion of novel depths. Our findings have important conservation implications for anthozoan lineages, as the loss of ‘source’ slope lineages could threaten millions of years of evolutionary history and confound future invasion events, thereby warranting protection.

## 1. Introduction

Determining how lineages invade novel habitats is fundamental to understanding the evolutionary processes underpinning global patterns of biodiversity. Colonization of novel habitats across deep time has led to high species diversity, the radiation of groups across wide bathymetric ranges and evolutionary success of lineages across the tree of life [1,2]. However, there is a lack of knowledge surrounding the mechanisms that facilitate lineage expansion into novel habitats, such as the evolutionary adaptations that precipitate invasion and how frequently these events occur, or the ancestral origins of these lineages [1,3,4]. These knowledge gaps are especially pronounced for groups with limited fossil records and lineages that occur across a wide range of biomes, such as shallow waters to the deep sea [5,6].

The deep sea was once thought to be devoid of life due to high pressure, near freezing temperatures and perpetual darkness – articulated by Edward Forbes' Azoic hypothesis [7]. However, the Challenger expedition of 1872 to 1876 collected a diverse fauna from the shallows down to depths of greater than 10 km in the Mariana Trench [8]. Since then, different mechanisms have been proposed to explain the origin and evolution of deep-sea biodiversity. For example, the onshore-to-offshore hypothesis suggests that disturbances including waves and storms at shelf depths (0–250 m) creates habitats that are challenging for species to inhabit, which results in diversification of species to occupy these habitats [9,10]. Compared with the shelf, the slope (250–3000 m) is exposed to fewer environmental perturbations, with little-to-no effect from waves and storms [11]. However, the slope is generally regarded as exhibiting greater topographic complexity (e.g. canyons, ridges and seamounts) and strong gradients in environmental conditions along depth gradients, which the depth-differentiation hypothesis suggests creates opportunities for species to evolve and occupy these diverse habitats [12,13].

The increased frequency of disturbances on the shelf and topographical complexity and strong environmental gradients with depth on the slope are both thought to drive divergence and phenotypic novelties (i.e. traits that are not present in ancestral species) that have facilitated adaptive radiations across a wide range of taxonomic groups [14–16]. Increasing taxonomic diversity, which is accompanied by an increase in the diversity of ecological traits, leads to incremental success forming deeper-occurring populations and species (the depth-differentiation hypothesis) [9,17–20]. The source-sink hypothesis describes how bathyal source lineages invade the abyss in a sink capacity, formed and regulated by a balance between immigration from the slope and extinction in the abyss via Allee effects [21]. Available nutrients and hard substrate generally decrease with depth, necessitating morphological adaptations and/or strategies to facilitate species persistence in challenging deep-sea habitats [22–24]. Diversification in the abyss is possible, demonstrated through discoveries of relict species at these depths [22–24]; however, catastrophic anoxic events in the abyss through deep time [25,26] have caused extinctions of abyssal species, leading to modern abyssal species representing younger lineages than their shallow-water counterparts [27].

Anthozoans (sea anemones and corals) have a long evolutionary history spanning the entire Phanerozoic and have colonized every marine habitat from the shelf to the abyss [28,29], and therefore provide an ideal model taxon to understand evolutionary invasion and persistence in novel habitats. Black corals (Hexacorallia: Antipatharia) are an anthozoan lineage with origins that can be traced back over 300 Ma [29] and occur across a wide range of habitats from the tropics to the poles and from surface waters to depths over 8000 m [30,31]. They are ecologically important because they provide habitat for many other species; for example, 2554 invertebrates were found living on a single black coral colony [32]. Black corals are also threatened in the deep sea via dredging, bottom trawling and extractive activities [33,34], and due to their slow growth rates, recovery from disturbances can take considerable time [35].

Despite their ecological and evolutionary importance, knowledge gaps remain regarding the evolutionary history of the group. Filling these knowledge gaps can lead to identification of the processes that drive bathymetric evolution of corals through deep time. Here, we determine the direction of evolutionary invasion into new depths and examine the evolutionary mechanisms that drove the diversification of black corals through deep time. We reconstruct a time-calibrated phylogeny based on target-capture enrichment of 2380 conserved loci (ultraconserved elements and exonic loci) [36] from 92 taxa (including outgroups: electronic supplementary material, table S1) and use a Dispersal-Extinction-Cladogenesis (DEC) model to estimate ancestral depth ranges to date the origin and trace the bathymetric evolution of black coral lineages.

## 2. Methods

### (a) Sample collection

Eighty-three black corals (ranging in depths from 14 to 4744 m, in all oceans from latitudes 57° N to 68° S) representing all seven families and 30 out of 45 accepted genera were chosen for this study because they occur at shelf (less than 250 m), slope (250–3000 m) and/or abyssal (greater than 3000 m) depths. Nine outgroups were also included, representing orders Actiniaria, Zoantharia, Scleractinia and Corallimorpharia, for time calibration purposes and to root the phylogeny. Seven of the 83 specimens included were published in Quattrini *et al*. [29], 24 from Horowitz *et al*. [37] and building upon these studies, we include targeted capture data for 52 new black coral specimens (electronic supplementary material, table S1). Specimens were collected by SCUBA, trawl, or via remotely operated vehicle and deposited in museums around the world. Specimen metadata are detailed in electronic supplementary material, table S1.

### (b) DNA extraction, library preparation and targeted enrichment

DNA was extracted using a Qiagen Puregene Tissue Kit following the DNA Purification from Tissue protocol. PCR inhibitors were removed from DNA using a Qiagen DNeasy PowerClean Clean Up Kit. A Qubit 2.0 fluorometer was used to measure the initial concentration of each extracted DNA sample and then the DNA was precipitated out, dried down and sent to Arbor Biosciences (Ann Arbor, MI) for library preparation, hybrid enrichment and sequencing, following details in Quattrini *et al*. [38]. The targeted enrichment of ultraconserved elements (UCE) and exonic loci was carried out using the hexacoral-v2 probe design, which includes 25 514 baits targeting 2499 loci [36]. Bioinformatic post-sequencing analyses were conducted following the Phyluce online documentation (https://phyluce.readthedocs.io/en/latest/tutorial-one.html), including raw read trim and matching of loci to UCE and exon probes. SPAdes v3.12.0 was used outside of the phyluce pipeline to assemble trimmed raw reads using the main executable script spades.py and a coverage cutoff of 2. Individually aligned UCE/exon loci were filtered to include only those that were present in at least 50% of the samples. All code used in this study are detailed in electronic supplementary material, Dataset S1.

### (c) Phylogenomic reconstruction and time calibration

IQtree v1.7 [39] with 1000 ultrafast bootstrap replicates was used to create a maximum-likelihood phylogeny. ModelFinder [40] was used to determine the best model scheme for each UCE/exon partition to infer the evolutionary relationships within the order Antipatharia. IQtree was also used to reconstruct 1063 individual bootstrap trees, one for each locus post-filtering 50% taxon occupancy, and a consensus tree. Newick utilities v1.6 [41] was used to remove low support branches (less than 30% bootstrap support), following the Astral III [42] online tutorial (https://github.com/smirarab/ASTRAL/blob/master/astral-tutorial-template.md). TreeShrink was used to remove outlier long branches from individual gene trees and corresponding gene alignments, following the online documentation (https://github.com/uym2/TreeShrink) [43]. IQtree was again used to reconstruct individual bootstrap trees from the cleaned alignments produced by TreeShrink, and then ASTRAL-III, a multi-species coalescent method, was used to estimate the resulting species tree [42] from the individual gene trees.

SortaDate [44] was used to identify the 25 most ‘clock-like’ loci (i.e. loci with properties of moderate length trees) from the set of 1042 loci, which were used for this analysis, as per Oliveros [45]. The maximum-likelihood phylogeny was used as a starting tree for time-calibration using BEAST v. 2.6.3 with four secondary calibration points selected from Quattrini *et al*. [29]; Zoantharia crown node (436 Ma, 95% highest posterior density (HPD) 336–531), Actiniaria crown node (513 Ma, 95% HPD 424–608), Scleractinia crown node (386 Ma, 95% HPD 324–447), the black coral crown node excluding the Leiopathidae (321 Ma, 95% HPD 249–407) and the root of the phylogeny Zoantharia + Actiniaria (642 Ma, 95% HPD 542–746) with normal distribution priors matching these HPDs. A relaxed clock model was used with a lognormal distribution on the ucld mean and uniform distribution on the ucld.stdev (initial 0.1, 0–1 bounds), as per Quattrini *et al*. [29]. A guide tree was used to ensure non-black coral nodes were congruent with studies that reconstructed time-calibrated phylogenies inferred from fossil calibrations including Quattrini *et al*. [29]. Two individual BEAST runs (see BEAST xml file in electronic supplementary material, Dataset S2) of 250 million generations were completed, with resulting log and tree files combined in LogCombiner [46] after the removal of 10% of generations as a burnin period. Tracer v. 1.7.1 [47] was used to assess convergence of parameter values and age estimates, and TreeAnnotator [46] was used to produce a maximum clade credibility tree using mean node heights.

### (d) Ancestral state reconstruction

A DEC model was implemented in RevBayes [48] to estimate ancestral states of depth ranges, following the DEC analysis online tutorial (https://revbayes.github.io/tutorials/biogeo/biogeo_simple.html). Expert opinions (Dennis Opresko, Tina Molodtsova and Marzia Bo) and the current literature were used to assign each taxon a depth range (shelf 0–249 m, slope 250–3000 m, abyss greater than 3000 m), or a combination of depth ranges for bathymetrically wide-ranging taxa (e.g. shelf-slope represents species occurring from 0 to 3000 m depth and slope-abyss from 250 to depths deeper than 3000 m) (see electronic supplementary material, table S2). A Markov chain Monte Carlo (MCMC) analysis produced a maximum clade credibility tree and ancestral states were plotted using plot_anc_states in R package RevGadgets. The ggtree package [49] was used to plot ancestral depth states on the time-calibrated tree, following code provided in McFadden *et al.* [50].

## 3. Results

### (a) Black coral evolution

We resolved the relationships among 30 of the 45 valid genera in the order Antipatharia, representing species that occur from just below the ocean surface to over 8000 m depth. Both maximum-likelihood (ML) (electronic supplementary material, figure S1) and multi-species coalescent (MSC) (electronic supplementary material, figure S2) analyses recovered congruent topologies with strong node support.

**Figure 1.**
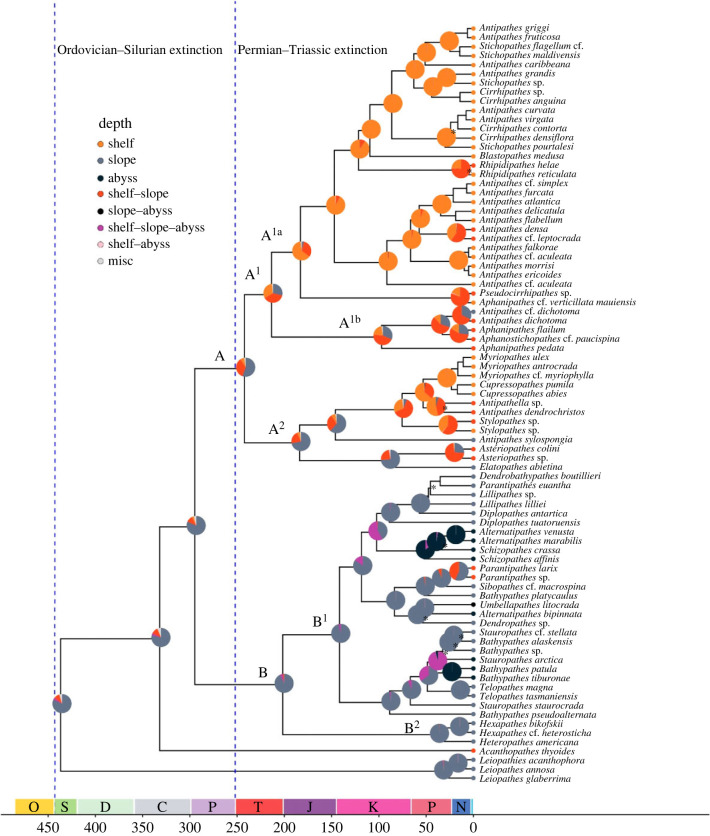
Time-calibrated phylogeny of the Antipatharia with ancestral character states of depth. BEAST2-dated phylogeny constructed from 25 clock-like loci. Depth ranges for each depth zone are as follows: shelf (0–250 m), slope (250–3000 m) and abyss (greater than 3000 m). Posterior probability values at each node are greater than 0.95 unless indicated by ‘*’. Tree is scaled to time in millions of years. Mass extinction events are shown (dashed vertical lines). Ancestral state reconstructions for depth are illustrated with pie diagrams at nodes; (O, Ordovician; S, Silurian; D, Devonian; C, Carboniferous; P, Permian; T, Triassic; J, Jurassic; K, Cretaceous; P, Palaeogene, N, Neogene).

Our time-calibrated phylogeny indicated that the black coral lineage diverged from the Scleractinia + Corallimorpharia approximately 601 Ma (95% HPD 483–719) (figure 1 and electronic supplementary material, figure S3). The phylogeny dates the black coral crown node to the Silurian period, approximately 437 Ma (95% HPD 325–567), at upper and middle slope depths (250–3000 m) (figure 1). At this time, the oldest extant family, the Leiopathidae, diverged from the rest of the order. A genus in the family Aphanipathidae, *Acanthopathes*, is the next lineage to diverge from all other families at 332 Ma (95% HPD 261–404) and occurred at shelf-slope depths (250–3000 m). The remaining lineages, which include 95% of extant black corals [51], diverged 295 Ma (95% HPD 222–366 Ma) during the Carboniferous-Permian to form two distinct clades (hereafter ‘Clade A’ and ‘Clade B’). The most recent common ancestor of Clade A and Clade B is estimated to have diverged 295 Ma (95% HPD 222–366) at a slope depth (82% pp). Clade A with a crown node of 242 My (95% HPD 145–310) consists of the families Antipathidae, Aphanipathidae, Myriopathidae and Stylopathidae, and Clade B with a crown node of 202 My (95% HPD 131–283) consists of the families Schizopathidae and Cladopathidae.

### (b) Bathymetric transitions

Clade A consists of two main clades; one of which (hereafter Clade A^1^) dated 213 Ma (95% HPD 150–280) likely occurred on the shelf and slope (proportions of estimated ancestral depths: 31% posterior probability (pp) on shelf, 29% pp on slope, and 38% pp on shelf-slope), and a second lineage (hereafter Clade A^2^) dated 184 Ma (95% HPD 110–261) likely occurred on the slope (72% pp). Clade A^1^ also consists of two main lineages (hereafter Clade A^1a^ and Clade A^1b^). Clade A^1a^ dated 183 Ma (95% HPD 99–202) likely occurred on the shelf (63% pp), representing an onshore transition with most subsequent lineages remaining on the shelf. While most of the taxa in Clade A^1a^ occur in shelf environments, there were three recent invasions into slope depths (occurring within the last 20 My) in this clade, although these three lineages also retained their shelf distributions and thus occur across a wide depth range. The crown of Clade A^1b^ was dated 97 My (95% HPD 35–180), and both crown and subsequent nodes within this clade likely occurred on the shelf and slope (45% pp shelf-slope, 31% pp slope and 21% pp shelf). This represented another onshore-directed bathymetric expansion from ancestors that occurred at slope depths. Most Clade A^2^ lineages remained on the slope for the past 184 My and have expanded their bathymetric ranges to also include the shelf. However, one lineage of Clade A^2^ invaded the shelf from slope depths (figure 1). In total, 27 out of 36 species in Clade A transitioned onshore to the shelf, representing at least five independent onshore transition events. After those onshore transitions, there were at least three independent bathymetric expansions to include the shelf and slope. Clade A^1^ represents a lineage that did not completely transition onshore to the shelf but does include species that now occupy both shelf and slope depths, with one taxon transitioning offshore to only occur on the slope.

Clade B consists of two main lineages hereafter referred to as Clade B^1^ and Clade B^2^. Clade B^1^, dated 141 Ma (95% HPD 95–198), and Clade B^2^, dated 37 Ma (95% HPD 14–76), both originated on the slope (estimated ancestral depths: 96% slope and 99% slope, respectively), suggesting that at these times, ancestral black corals occurred at roughly the same depths as their ancestors. Clade B^1^ diverged into two lineages: one lineage consisting of extant slope taxa and abyssal taxa representing at least one distinct offshore transition, and the other lineage consisting of shelf-slope, slope, slope-abyssal and abyssal taxa, representing a distinct broadening of bathymetric ranges both onshore and offshore from their common ancestor in the past 80 My. Clade B^2^ comprises extant slope and abyssal taxa, indicating that these lineages either stayed at slope depths or transitioned offshore to the abyss. In total, eight ancestral antipatharian lineages from Clade B remained on the slope and at least one transitioned to shallower habitats (shelf-slope) over the last 200 My. Within the last 50 My, at least four independent lineages have expanded to, or completely transitioned to, the abyss.

## 4. Discussion

### (a) Slope origin of black corals

Our phylogenomic reconstruction traces the origin of black corals to the Ediacaran Period (601 Ma) and first diversification of this group to the early Silurian (437 Ma). The earlier origin in this study compared with a recent assessment of Anthozoa [29], which dated the origin of the Antipatharia to the Cambrian Period (522 Ma) and first diversification to the Carboniferous (321 Ma), is due to our inclusion of the monogeneric family Leiopathidae. Leiopathidae is the first lineage to branch off from the Antipatharia and is sister to all other black corals (figure 1). The diversification of Antipatharia falls just after the Great Ordovician Biodiversification Event (485 to 443 Mya), which gave rise to suspension feeding metazoans that possessed the ability to consume highly diverse zooplankton in the water column [52,53]. Based on the fossil record, filter-feeding taxa such as black corals came to dominate benthic marine ecosystems for the remainder of the Paleozoic Era [53,54].

Our ancestral reconstruction supports black corals' first diversification at slope depths. Additionally, most extant species occupy shelf and slope habitats between 50 and 800 m [55], with less than 20 species occupying the abyss. Based on our reconstruction, all abyssal lineages originated within the last 50 My. The habitat heterogeneity and topographical complexity of the upper slope has been linked to increased rates of species formation [12,13] in groups including octocorals [19], bivalves [12] and brittle stars [56]. Our results lend support to the depth-differentiation hypothesis, which could explain increased species diversity of black corals in slope depths.

Reconstructions of ancestral antipatharians can only be inferred from lineages of extant species and from the very limited fossil record of the group [57,58]. Two genera and three species of shallow water black coral fossils have been described from shelf depths during the Lower Ordovician (∼470 Ma) Fenxiang Formation of Hubei Province in southern China [57,58]. These fossil records were not included in our phylogenetic and bathymetric reconstructions because of morphological differences between the fossils and extant black corals, and uncertainty regarding whether the fossils represent lineages that share a direct common ancestor with present-day species [59]. Nevertheless, our divergence dating results correspond well with these putative black coral fossils and given that the slope has been a more stable environment across geological time than the shelf [11], it is possible that earlier antipatharians occupied shelf depths before going extinct.

### (b) Onshore transitions and morphological innovations

Based on our reconstruction, ancestors of Clade A^1^ transitioned onshore to the shelf ∼183 Ma, coinciding with the early Jurassic reef crisis (183 Ma). During this period, reef-building corals with calcium carbonate skeletons were negatively affected by large-scale volcanism, global warming and increased atmospheric pCO_2_ [29,60,61]. This event might have vacated niches or reduced competition for black corals and other non-calcifying groups (e.g. octocorals) to invade the shelf [29,62]. Most black corals in Clade A^1^ have since diversified in the more dynamic shelf environment [9,10] potentially driving a rapid radiation in the group. We do not explicitly test rates of diversification here due to the limited sampling of species; however, there is evidence of elevated diversity on the slope: over 20 extant black coral genera occur on the slope, including the genus *Antipathes* that currently contains 75 of 300 currently accepted species in the order [51].

**Figure 2.**
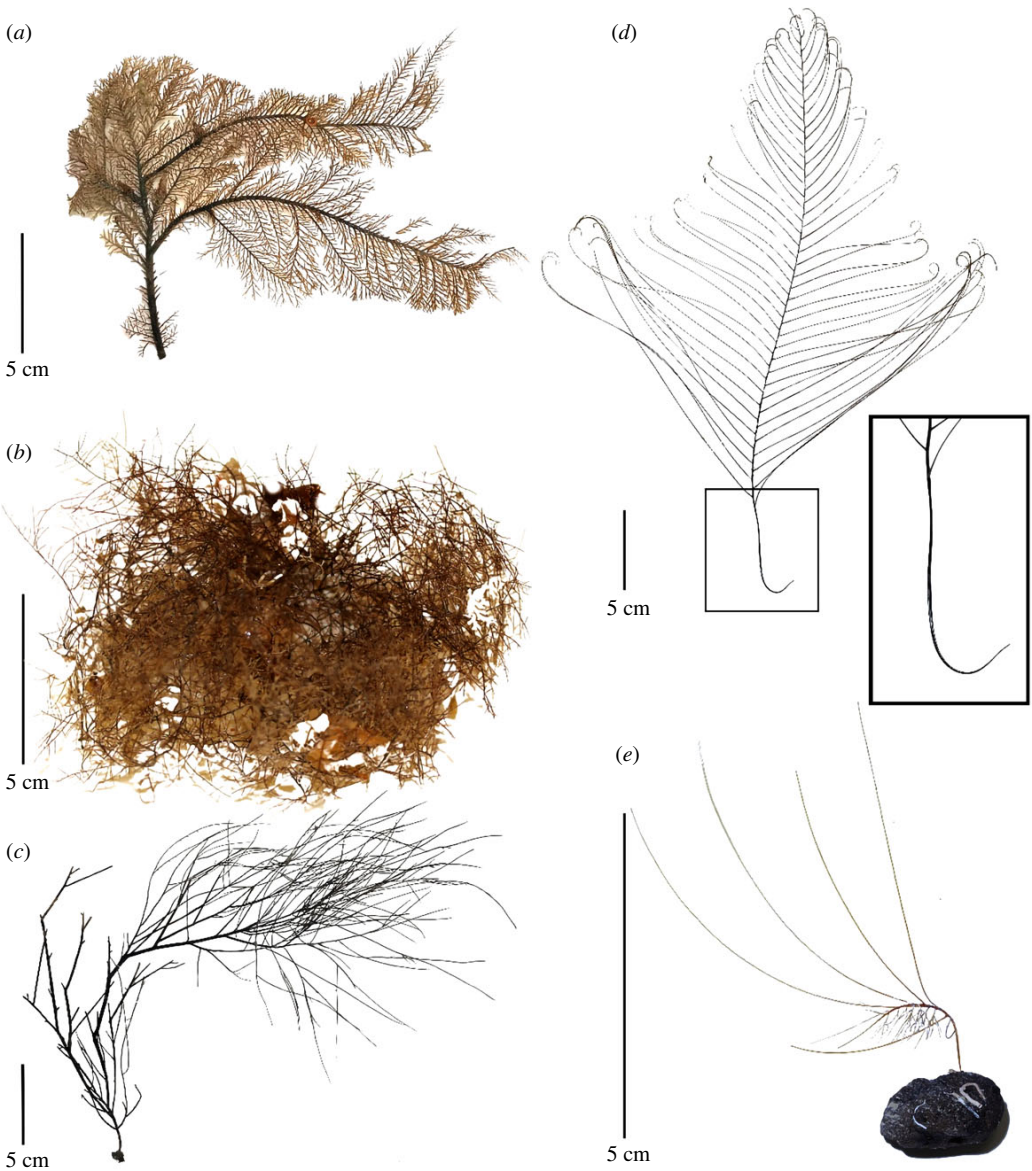
Morphological features of the Antipatharia. *Myriopathes* sp. colony showing flabellate and pinnulate characteristics (*a*), *Antipathes* sp. colony showing bramble and non-pinnulate characteristics (*b*), *Antipathes* sp*.* colony showing branched and non-pinnulate characteristics (*c*), *Schizopathes affinis* showing pinnulate branches and a basal hook, enlarged in inset (*d*), and *Heteropathes americana* showing a curved stem and pinnulate branches that form a wind-tunnel appearance (*e*).

Species in both Clade A^1^ and Clade A^2^ invaded into, then diversified at shelf depths; however, shelf invasion occurred much later for Clade A^2^ (48 Ma versus 183 Ma). Given that Clade A^1^ invaded at a time when competition on the shelf was potentially lower, they may not have required morphological adaptations to persist in their new environment. By contrast, delayed invasion by Clade A^2^ might have facilitated the development of morphological adaptations. Unlike species in Clade A^1^, species in Clade A^2^ are pinnulate, increasing their surface area and enhancing their capacity for heterotrophic feeding (figure 2*a*). While pinnulate morphology may present challenges in environments with high hydrodynamic energy due to increased surface tension and friction [63], pinnulate species are potentially better adapted to low-energy, high-turbidity environments where their greater surface area allows for more efficient nutrition uptake despite low rates of nutrition availability. This apomorphic trait may have facilitated ecological divergence from non-pinnulate black corals and enabled the expansion of species ranges to persist in both sheltered shelf and slope habitats.

### (c) Offshore transition and morphological innovations

The ancestors of Clade B likely occurred at slope depths, and only one lineage from this clade (*Parantipathes* spp.) expanded in an onshore direction to also occupy the lower shelf (minimum depth of ∼200 m). All other lineages in this clade have remained on the slope, but in the past 50 My at least five independent slope lineages in Clade B (containing about 30 species) invaded the abyss. This suggests that extant lineages of black corals only invaded into and diversified at abyssal depths relatively recently, supporting the source-sink hypothesis of abyssal species. However, the question remains: why are there not older lineages currently occupying the abyss?

Invertebrate lineages have historically and routinely invaded the abyss and subsequently gone extinct, either due to minimum viable population sizes in large abyssal habitats [64] or to repeated anoxic events [12,25,26,65]. The most recent anoxic event in the abyss occurred during the earliest Palaeocene 66 Ma, immediately following the K-T extinction event [17,66]. This global anoxic event, combined with limited nutrition availability and hard substrate required for feeding and settlement, could have eradicated abyssal black coral lineages present in the Cretaceous. The return of habitable conditions in the abyss in Palaeogene [17] may have allowed contemporary black coral lineages to invade abyssal habitats. However, diversification into a novel depth zone with physiologically challenging conditions requires key morphological and physiological innovations. Thus, for lineages that persisted on the slope for millions of years, morphological and/or physiological adaptations would be necessary to invade and survive in the abyss [67].

Abyssal black corals are morphologically different than shelf and slope taxa. Abyssal black corals have simple branching characteristics, all of which are pinnulate (figure 2*d–e*), a feature that increases surface area and enhances the ability of a coral to filter-feed in a low-nutrient environment. By contrast, shelf and slope taxa can be either pinnulate or non-pinnulate and exhibit a wide variety of branching characteristics including flabellate, bramble, contorted and irregularly branched (figure 2*a–c*). Furthermore, all shelf and slope species have basal plates that allow a colony to attach to hard substrate (figure 2*d*) while the strictly abyssal genus *Schizopathes* has basal hooks (derived from ancestral basal plates) that allows settlement on extremely small pebbles or rocks (figure 2*d*), or possibly just sand or mud. Shelf and upper slope species also generally possess upward directed stems, while some lower slope and abyssal species have distinct stems bent at a 90° angle and curved pinnules that resemble wind tunnels. These features position the stem parallel to the substrate, pivot in changing current directions and funnel nutrients through the colony, thereby maximizing contact of nutrients and the polyps (figure 2*e*). Pinnulation, basal hooks and wind-tunnel characteristics could represent independent abyssal adaptations and/or exaptations that enabled black corals to invade and survive in the abyss.

These morphological adaptations also enabled the persistence of abyssal lineages by limiting interspecific competition and ecological divergence to avoid competitive exclusion [20,68–70]. Basal hooks enable colonies to settle and grow in sandy habitats isolated from sister lineages that require hard substrate for settlement, thereby isolating gene pools to promote diversification and obtain nutrients in habitats with limited competition [20]. Species that possess a wind-tunnel morphology can persist in the lowest-nutrition environments, providing an advantage over other abyssal species that require locations with higher nutrient levels.

Ancestral state reconstructions can be influenced by incomplete taxon sampling [71]. Although additional transitions could potentially be detected with the inclusion of more taxa, our major conclusions regarding the origin and the direction of lineages' invasions are unlikely to change. First, our analysis includes three of the four deepest-known black coral genera [72], lacking only *Abyssopathes*. If *Abyssopathes* were included in this analysis, it would further support a recent invasion from the slope to the abyss (in Clade B^2^). In addition, there are 12 slope genera, three shelf genera and an unknown number of extinct lineages that were not included in this study. Inclusion of these lineages would unlikely change the results of the ancestral reconstruction as most missing extant genera are found in depths similar to other species in their respective families.

### (d) Evolutionary refugia

Understanding the evolutionary history of a group of species provides insight into the mechanisms that have enabled their persistence through deep time. These insights can help us predict outcomes from threats and identify priority areas for conservation. Bathymetrically wide-ranging taxa are threatened by a variety of anthropogenic activities, including increased storm activity, fishing pressure and sea-level changes in shelf habitats, and resource extraction activities (oil/gas, fishing and future mining) in deeper waters [73]. The extinction of shallow and deep lineages would likely have long legacy effects on marine biodiversity because they are important foundation species in marine ecosystems. Our results also suggest that it would take millions of years for these taxa to be replenished, as indicated in ancestral state reconstructions and divergence dating of the phylogeny through deep time; therefore, it is critical to preserve these lineages under looming threats of ocean change and anthropogenic activities. Our results also show that continental slope lineages have evolved and diversified into a variety of habitats from shallow waters to the deep abyss and also serve as ancestral lineages to shallow- and deep-sea species. Therefore, additional protection for continental slope taxa, some of which are the oldest animals on the planet (colonies are slow growing and can live ∼4,000 years, [74]), may be warranted due to their potential role as evolutionarily refugia in the face of long-term global ocean change. Although studies of evolutionary history such as this cannot predict future outcomes, understanding the patterns of evolution through the deep past can help to pinpoint origins of diversification and thus lineages in need of protection, which is particularly important for sentinel, foundation species, such as corals.

## 5. Conclusion

Our time-calibrated phylogeny indicates that black corals diversified at slope depths ∼437 Mya, and radiated bidirectionally, first onto the shelf and much later into the abyss, rather than in a unidirectional onshore-offshore pattern. Bidirectional radiation of lineages has also been found in other marine lineages [25,56,75,76]; however, for most cnidarians this has yet to be formally investigated. Ancestral state reconstruction suggests that morphological adaptations have influenced the invasion and persistence of black corals in different habitats through deep time, a finding consistent with other marine lineages [77–80]. Our study also indicates that abyssal lineages are younger than slope and shelf lineages, therefore supporting the source-sink hypothesis that abyssal taxa originate from slope habitats [21]. In addition, our results support the depth-differentiation hypothesis as lineages were found to originate and diversify across the continental slope. Therefore, our results underscore the importance of understanding evolutionary history for both explaining modern-day patterns of marine biodiversity across depth and predicting the consequences of ongoing environmental change. Additionally, our findings emphasize the role of habitats that have ‘sources’ of anthozoan and other marine lineages, which further promote diversification. Loss of this phylogenetic diversity would threaten millions of years of evolutionary history; and therefore, it is important to identify and prioritize conservation resources to protect these habitats, and to limit extinction across the tree of life [81].

## Data Availability

Raw data is available in The National Center for Biotechnology Information (NCBI) (https://www.ncbi.nlm.nih.gov/). Bioproject and Biosample reference identifications are listed in electronic supplementary material, table S2. Raw data for 24 specimens are available on Dryad Digital Repository: https://doi.org/10.5061/dryad.h44j0zprt [82] (see supplementary material, table S2). The data are provided in electronic supplementary material [83].
